# OUTCOMES OF SURGICAL TREATMENT OF DIAPHYSEAL FEMUR FRACTURES IN POLYTRAUMATIZED CHILDREN

**DOI:** 10.1590/1413-785220243205e278518

**Published:** 2024-11-01

**Authors:** David Ken Nagata Radamessi, Douglas Rulo de Nicola, Lucas Pereira Sarmento, Ana Luisa Bertollo De Prá, Natália Medeiros Mourão, Luiz Fernando Cocco, Eiffel Tsuyoshi Dobashi

**Affiliations:** 1. Universidade Federal de São Paulo, Escola Paulista de Medicina, Departamento de Ortopedia e Traumatologia, São Paulo, SP, Brazil.; 2. Centro Universitário Brasileiro Multivix, Vitoria, ES, Brazil

**Keywords:** Child, Child Hospitalized, Femoral Fractures, Fracture Fixation, External Fixators, Intramedullary Fracture Fixation, Criança, Criança Hospitalizada, Fraturas do Fêmur, Fixação de Fratura, Fixadores Externos, Fixação Intramedular de Fraturas

## Abstract

Objective: This study aimed to evaluate the surgical treatment of diaphyseal femur fractures in polytraumatized children, considering consolidation rate, complications, and function as outcomes of interest. Methods: This is a quantitative, cross-sectional, and retrospective study of medical records of patients treated from 2012 to 2021. The 39 patients (41 femurs) were divided into four groups according to the method of osteosynthesis. We used the IBM SPSS Statistics software, version 20 (SPSS Inc, Chicago, IL), and performed Chi-square, Fisher, Kruskal-Wallis, and Shapiro-Wilk tests with a significance level of 5%. Results: We observed six complications among patients. Functional outcomes were satisfactory in 40 cases (97.6%) and unsatisfactory in one case (2.40%) according to the adopted criteria. We found one case (2.40%) of non-union and one case (2.40%) of malunion, whereas 39 cases (95.20%) achieved full consolidation. Conclusions: Flexible intramedullary nails and external fixators are the preferred options for patients aged 5 to 10 years. Intramedullary nails, plates, or external fixators are prioritized for patients over 11 years old. The type and pattern of fractures were considered relevant for treatment selection. ***Level of evidence III, Therapeutic study - Investigation of outcomes and treatment. Comparative retrospective study.***

## RESUMO

Objetivo: Este trabalho teve como objetivo avaliar o tratamento cirúrgico das fraturas diafisárias do fêmur, em crianças politraumatizadas, considerando os desfechos: taxa de consolidação, complicações e função. Métodos: Trata-se de estudo quantitativo, transversal, retrospectivo de prontuários de pacientes atendidos entre 2012 e 2021. Os 39 pacientes (41 fêmures) foram divididos em quatro grupos de acordo com o método de osteossíntese. Utilizamos o *Software IBM SPSS Statistics* , versão 20 (SPSS Inc, Chicago, IL) e os testes do Qui-quadrado, Fisher, Kruskal-Wallis e Shapiro-Wilk com significância de 5%. Resultados: Obtivemos seis complicações entre os pacientes. O resultado funcional foi satisfatório em 40 (97,6%) situações e insatisfatório em uma (2,40%) de acordo com os critérios adotados. Tivemos um (2,40%) pseudartrose e uma (2,40%) consolidação viciosa; 39 (95,20%) casos obtiveram plena consolidação. Conclusões: As hastes intramedulares flexíveis e o fixador externo são as opções preferenciais para pacientes com idade de 5 a 10 anos. As hastes intramedulares, placas ou fixadores externos são prioritários para pacientes com mais de 11 anos. O tipo e o padrão das fraturas foram considerados relevantes para a escolha do tratamento. ***Nível de evidência III, Estudo terapêutico – Investigação dos resultados e tratamento. Retrospectivo comparativo.***

## INTRODUCTION

 Treatment choice for diaphyseal femur fractures (DFF) in the pediatric age group depends on the age, size, and pattern of injury in the patients involved ^1^ . 

 Success depends on the careful selection of methods to achieve the best possible outcomes. Surgery is generally indicated for the following clinical situations: fractures associated with multiple injuries, such as polytrauma, multiple fractures, floating knee, open fractures, fractures associated with extensive skin and soft tissue injuries, pathological fractures, and fractures associated with head injuries. ^2^

 The socioeconomic conditions of the family and the treatment environment are also important factors guiding treatment choices, in addition to the cost of treatment and the availability of different types of osteosynthesis. The patients’ caregivers usually work, and their absence from work can result in undesirable financial burdens. Prolonged absence from school can also affect the educational needs of the children. ^3^

 Surgical treatment using osteosynthesis falls into three modalities: plate and screws, external fixation, and intramedullary fixation. The use of these devices has been shown to prevent many complications that can be caused by prolonged immobilization with casts, as well as psychological and financial damage due to extended hospital stays. ^3^

 The use of plates and screws is indicated for the treatment of comminuted fractures in which the maintenance of bone length is compromised, thus considered unstable, or in children with a body weight exceeding 49 kg. The intrinsic benefits of this modality include a lower rate of malunion, greater axial and torsional stability when the body weight is placed on the operated lower limb, and reduced exposure and less muscle damage when the submuscular technique is used. Some authors advocate the use of bridge technique for comminuted and unstable fractures to maintain or restore femoral bone length. ^4^

 On the other hand, the use of these implants by traditional methods shows some disadvantages as they require extensive exposure, causing more damage to the soft tissues surrounding the fracture. ^4^

 In recent years, flexible intramedullary nailing has become the preferred method for osteosynthesis, initially developed in Nancy, France. With the introduction of this method, many advantages have been recognized, such as the use of minimally invasive approaches, shorter hospital stays, early mobilization and rehabilitation, and a lower rate of complications. Its indication is for stable length DFF patterns, such as transverse and short oblique fractures, and can be performed by retrograde or antegrade insertion. This approach avoids osteonecrosis of the femoral head and premature closure of the growth plates, especially the greater trochanter, when trochanteric insertion nails are used. There are also rigid Ender nails that can be used to prevent shortening at the fracture site. However, complications such as skin irritation due to the prominence of the distal ends of the nails and migration due to premature implant loosening have also been reported. ^5^

 The use of external fixation methods offers several advantages. They are simple to apply, quick to install, and allow for temporary or permanent treatment of femoral fractures in patients kept in an intensive care environment. However, it is reported that complications associated with external fixation are more common, especially in children with multiple injuries. ^6^

 When researching the literature, there is a scarcity of studies comparing the advantages and disadvantages of different surgical treatment methods in terms of consolidation rates, complications, and functional outcomes. ^7^

 When comparing different methods of surgical stabilization, a systematic review by Madhuri et al. ^8^ found three trials that showed that the compiled studies were of low quality in determining the rate of complications such as malunion, serious adverse events, time to return to school, parental satisfaction, and children’s satisfaction in fractures treated with elastic intramedullary nails versus external fixation. The rates of serious adverse events and time to full weight-bearing in patients treated with dynamic versus static external fixation were also inconclusive. The authors found very low-quality evidence when comparing intramedullary fixation and submuscular plating regarding differences in malunion rates, serious adverse events, and time to weight-bearing. ^1^ ^,^ ^9^

 In the association between pediatric DFF and polytrauma, it is known that injuries to the head, chest, and abdomen can occur. The Waddell triad is described in association with high-energy kinetic injuries, such as high-speed motor vehicle accidents or pedestrian accidents. In healthy children, there can be an acute loss of blood volume of approximately 25 to 30% before changes in blood pressure can be recognized, resulting in hypovolemia and a potential state of shock. A child with DFF and hypotension should be carefully investigated for bleeding. ^6^

 Thus, the ideal treatment for femoral fractures in polytrauma depends strictly on the child’s age and severity of other injuries. Within the principles of care, there is evidence suggesting that early surgical stabilization of fractures results in a lower rate of complications due to shorter periods of ventilatory support, intensive care unit stays, and hospitalization. ^10^

Based on the above, this study aimed to evaluate the surgical treatment of DFF in polytraumatized children using four different therapeutic stabilization methods, considering outcomes such as consolidation rate, complications, and function.

## MATERIALS AND METHODS

This is a quantitative, retrospective cross-sectional study based on the review of medical records of patients diagnosed as polytraumatized with diaphyseal femur fractures (DFF) in the pediatric age group who were treated at the Hospital Geral de Pirajussara (a reference center for the municipalities of Embu das Artes and Taboão da Serra, São Paulo, Brazil, operating under the Brazilian Unified Health System - SUS) from January 2012 to December 2021.

The study was developed as an extension of a project previously approved by the Research Ethics Committee (CEP) of the Federal University of São Paulo - UNIFESP, with Ethical Review Presentation Certificate number 59474522200005505, and approved for execution under the CAAE number 5.612.632. The procedures followed the guidelines of the UNIFESP Research Ethics Committee for experiments involving human subjects, in accordance with the Helsinki Declaration of 1995.

Inclusion criteria were:

- Patients of both sexes.
- Age from 2 to17 years.
- Patients undergoing surgical treatment.
- Diagnosed with polytrauma and DFF.
- Simple, complex, closed, or open fractures.
- Complete medical records.
- Minimum outpatient follow-up = outpatient discharge or six months.
- Signing an informed consent form.

Meanwhile, exclusion criteria were:

- Patients with pathological fractures.
- Femoral fractures in other segments.
- Incomplete medical records.
- Failure to sign an informed consent form.

The patients were divided into four groups based on the osteosynthesis method used for fracture treatment. Group A consisted of 13 (31.70%) patients treated with linear or circular external fixators; Group B included six (14.63%) patients treated with plates and screws; Group C included 13 (31.71%) patients treated with intramedullary fixation. These were subdivided into C1 (blocked intramedullary nail) with nine (21.95%) and C2 (flexible intramedullary nail) with four (9.76%). Group D comprised nine (21.95%) patients who initially underwent surgical treatment with an external fixator and later converted to internal fixation.

 Thus, after applying the eligibility criteria, 39 patients with DFF were included for analysis. Cases with bilateral fractures were considered individually (N=2). Thus, the overall sample size of cases evaluated was 41 operated femurs. Of the total, 11 (28.21%) patients were female, and 28 (71.79%) were male. Table 1 provides an overview of the sample, considering age and sex. Epidemiological characterization was also conducted for individual cases ( Table 1 ). 

**Table 1.** Characterization of the overall sample and by fixation method by age and sex (N=41)

	Total		Sexo			
		Male		Female		
	N (%)	Age (years) Mean (SD)	N (%)	Age (years) Mean (SD)	N (%)	Age (years) Mean (SD)

Overall sample	41	11,8 (5,01)	28 (68,29)	12,54 (4,62)	13 (31,7)	10,23 (5,61)
Fixator*	13 (31,7)	6,85 (5)	8 (19,51)	8,13 (4,76)	5 (12,2)	4,8 (5,17)
* Circular external fixator	1 (2,44)	15	1 (2,44)	15	0 (0)	
*Linear external fixator	12 (29,27)	6,17 (4,55)	7 (17,07)	7,14 (4,18)	5 (12,2)	4,8 (5,17)
Plate	6 (14,63)	12,17 (2,93)	5 (12,2)	12,2 (3,27)	1 (2,44)	12
Locked nail	9 (21,95)	16 (1,41)	7 (17,07)	16 (1,53)	2 (4,88)	16 (1,41)
Flexible nail	4 (9,76)	11,75 (5,12)	3 (7,32)	12 (6,24)	1 (2,44)	11
Method conversion	9 (21,95)	14,56 (1,51)	5 (12,2)	15,4 (0,55)	4 (9,76)	13,5 (1,73)

1- For categorization purposes, the overall group included both circular and linear external fixators

A spreadsheet was created to tabulate relevant information collected in our sample, including sex, age at the time of the accident, fracture type, fracture pattern, treatment type, consolidation time, post-recovery function, and postoperative complications.

 Injuries were classified according to fracture pattern as simple (transverse, oblique, or spiral) and complex (wedge, segmental, and comminuted), and as closed or open, following the Gustillo and Anderson classification. ^11^

The consolidation process of injuries and function after therapeutic intervention were also assessed. Good outcomes were defined for individuals who exhibited complete fracture consolidation, a normal range of joint motion, normal muscle tone, grade V strength, absence of pain, limb length discrepancy less than 2 cm, and proper gait as determined by visual analysis. A poor outcome was defined if any of these criteria were not met. Sequelae and complications were analyzed in each of the studied groups, being subdivided into major (refracture, malunion, deep infection, non-union, shortening greater than 2 cm, limited joint range of motion, and/or infection such as osteomyelitis and septic arthritis) and minor (pin tract infection, screw breakage, nail migration, and/or implant-related tendinitis).

The groups were compared based on age, fracture type and pattern, consolidation success, functional analysis (using criteria adopted by the researchers), and complications, checking for statistical significance when possible.

 The data obtained were analyzed by a specialized medical statistician using the IBM SPSS Statistics software version 20 (SPSS Inc, Chicago, IL). Categorical data were presented in the form of frequencies and percentages, and continuous numerical data were presented as means and standard deviations. For categorical data, the Chi-square test and Fisher’s Exact test were used. The Kruskal-Wallis test was applied for non-homogeneous and non-parametric numerical data comparisons. Analyses were conducted to determine whether there was a statistical association between the different methods of surgical treatment for the studied variables, with a significance level of 5%. Normality of the variables was tested using the Shapiro-Wilk test, assuming non-normality when the result was p < 0.05. Confidence interval was set at 95%. We used 2×N tables to assess the association between treatments and other variables. The Cramer’s V coefficient was applied to evaluate the association between non-square tables. To specifically iscriminate statistical differences within the groups for the evaluated categories, adjusted residuals were analyzed (the difference between observed and expected frequencies adjusted according to the Z-score for Fisher’s Exact test, with a reference value of 1.96, corresponding to a significance level of 5%). Residuals > 1.96 or < 1.96 were considered statistically significant. ^12^

## RESULTS

For cases of method conversion (Group D), there was a need for conversion from external fixation to intramedullary nail (IN) in seven situations. Among these, six conversions from external linear fixator to nail occurred. In one of these patients, delayed consolidation occurred, with removal of the synthesis material being performed after 2.5 years. In another case, a strategic change from a plate to an IN was made due to loss of reduction. Additionally, there was one instance in which conversion from external linear fixator to bilateral plates was required. Finally, in yet another of these cases, although the osteosynthesis method was not changed, a reoperation was necessary to perform a femoral wedge osteotomy due to malunion, a complication associated with a reduction in the range of motion of the ipsilateral knee.

 In the comparison between groups, according to different fixation systems versus age, the Kruskal-Wallis test demonstrated an effect of the group on age [X²(4) = 22.315; P < 0.001], indicating a statistical difference in age among the groups ( Table 2 ). In order to identify which groups presented a statistically significant difference, pairwise comparisons were made for the groups considering the age of the participants. Pairwise comparisons showed a statistically significant difference between the “fixator-flexible nail” group (P = 0.019) and the “fixator-locked nail” group (P < 0.001). The others did not show significant differences considering the age of the samples. It should be noted that sample size (N) was higher for groups B, C, and D, accounting for 31.7%, 21.95%, and 21.95% of the overall sample, respectively. 

**Table 2.** Comparison of age versus fixation method (N=41)

	df	Chi-square (X²)	P-value*
Age vs. Fixation method	4	22,315	<0,001

2 - *Kruskal-Wallis test

Pairwise comparisons showed a statistically significant difference between the “fixator-conversion” groups (P = 0.019) and “fixator-locked nail” groups (p < 0.001). The other comparisons did not show significance for age.

Table 3 shows the comparisons between the groups and fracture patterns, consolidation, function, and complications (we marked YES or NO for positive cases among major and minor complications). When evaluating the consolidation category, we excluded two patients, one due to transfer to a private healthcare service and the other due to loss of follow-up. Only one case (in which there was a conversion from plate to nail due to loss of reduction) was considered a consolidation failure. Regarding complications, we found six positive situations, with three classified as major (malunion, limitation of joint range of motion, and pyoarthritis) and three as minor (pin tract infection, screw breakage in the nail without compromising consolidation or function, and secondary tendinitis at the implant insertion site). We highlight that 50% of the complication cases (N = 3) occurred in Group D, with two major complications (limitation of joint range of motion and pyoarthritis) and one minor (tendinitis). In Group A, we found one minor complication (pin tract infection) and one major complication (malunion); in Group C1, we found one minor complication (screw breakage in the nail). 

The functional outcome was satisfactory in 40 cases (97.6%) and unsatisfactory in one case (2.4%) according to our criteria. Regarding fracture consolidation, we found one case (2.4%) of non-union and one case (2.4%) of malunion. In total, 39 cases (95.2%) achieved full and satisfactory consolidation of their injuries.

 For the analyses between the studied groups, only comparisons between fracture pattern (simple vs. complex) and fracture type (open Gustillo 3A vs. closed) showed statistical significance. The Fisher’s Exact Test showed an association between fracture pattern and fixation method (X²(4) = 13.232; P = 0.007) and between fracture type and osteosynthesis type (X²(4) = 7.361; p = 0.029), with no association for comparisons of consolidation, function, and complications with p > 0.05 ( Table 3 ). 

**Table 3.** Comparison of fracture, consolidation, function, and complications versus fixation method (N=41)

		Fixation method N (%)					p-Valor*	Cramer’s V % (p-value)
		Group A	Group B	Group C1	Group C2	Group D		
Fracture type (N=41)	Open	0 (0)	0 (0)	0 (0)	1 (2,4)	3 (7,3)	0,029ᵃ	48,8 (0,045)
	Closed	13 (31,7)	6 (14,6)	9 (22)	3 (7,3)	6 (14,6)		
Fracture pattern (N=41)	Simple	9 (22)	2 (4,9)	5 (12,2)	3 (7,3)	0 (0)	0,007ᵇ	55,3 (0,014)
	Complex	4 (9,8)	4 (9,8)	4 (9,8)	1 (2,4)	9 (22)		
	Total	13 (31,7)	6 (14,6)	9 (22)	4 (9,8)	9 (22)		
Consoli dation (N=39)	Yes	12 (30,8)	6 (15,4)	8 (20,5)	4 (10,3)	8 (20,5)	0,692	29,6 (0,490)
	No	0 (0)	0 (0)	0 (0)	0 (0)	1 (2,6)		
	Total	12 (30,8)	6 (15,4)	8 (20,5)	4 (10,3)	9 (23,1)		
Function (N=31)	Normal	11 (35,5)	5 (16,1)	5 (16,1)	4 (12,9)	5 (16,1)	0,645	37,3 (0,366)
	Sequela	0 (0)	0 (0)	0 (0)	0 (0)	1 (3,2)		
	Total	11 (35,5)	5 (16,1)	5 (16,1)	4 (12,9)	6 (19,4)		
Complications (N=33)	Yes**	2 (6,1)	0 (0)	1 (3)	0 (0)	3 (9,1)	0,389	38,5 (0,298)
	No	10 (30,3)	5 (15,2)	4 (12,1)	4 (12,1)	4 (12,1)		
	Total	12 (36,4)	5 (15,2)	5 (15,2)	4 (12,1)	7 (21,2)		
Yes** (N=6)	Major	1 (16,7)	0 (0)	0 (0)	0 (0)	2 (33,3)	1	47,1 (0,513)
	Minor	1 (16,7)	0 (0)	1 (16,7)	0 (0)	1 (16,7)		

*Fisher’s exact test

a = Adjusted residues indicate a statistical difference in the “method conversion” comparison

b = Adjusted residues indicate a statistical difference in both the “method conversion” and “fixator” comparisons

For specific discrimination of statistical differences between treatment groups for the evaluated categories, adjusted residuals were analyzed. The number of individuals with open and/or complex fractures in the “Method Conversion” group was higher than expected (observed frequency 3 and expected 0.9 with adjusted residual = 2.7; and observed frequency 9 and expected 4.8 with adjusted residual = 3.2, respectively), and the number of complex fractures in Group A was lower than expected (observed frequency = 4 and expected = 7, with adjusted residual = −2). The adjusted residuals did not show a statistically significant difference between observed and expected frequencies for the other cells.

## DISCUSSION

 The definitive treatment of diaphyseal femur fractures (DFF) in the pediatric population remains undefined in the literature. This assertion is based on studies in which the levels of scientific evidence attained are still not ideal. ^2^ We observed that management is primarily based on the patient’s age, although skeletal age, pattern and type of injury, child size, and the expertise of the medical team are also considered determining factors for therapeutic choice. ^14^

 Our study showed a direct association between the age of the children involved and the method of fixation, corroborating the hypothesis predicted in numerous previous studies. ^13–15^

 We believe that there are numerous advantages associated with surgical treatment of femoral fractures, especially in polytraumatized individuals who require very specific care. Among the benefits of this modality are ease and early patient mobilization, improved respiratory capacity, prevention of bedsores, minimization of the risk of pulmonary infection, appropriate stabilization, reduced hospitalization periods, lower therapeutic costs, ease of hygiene, and mobility. ^11^

 Moreover, the superiority of surgical treatment has been reported for this population when compared to conservative methods. We noted that this theme has been the subject of discussion by various authors. ^7^ ^,^ ^11^ ^,^ ^16^ Regarding the various aspects of treatment with plaster casts, we can mention knee joint stiffness, malunion, shortening, delayed consolidation, refracture, muscle hypotrophy, and weakness, among others. ^17^

In our view, another relevant and significant aspect of this topic is the socioeconomic factors, such as the length of in-hospital stay, the burden on the institution, the impact on the family budget, absence from school, absence of parents or guardians from work, and job loss.

 Other issues have also been reported, such as transportation difficulties, hygiene problems, reduced social contact, total dependence for feeding care, psychological changes, and restricted visual field. ^17^

Our services are provided in a public referral facility for more complex treatments in the municipalities of Embu das Artes and Taboão da Serra, São Paulo, Brazil, which are affiliated with the Brazilian Unified Health System (SUS). Thus, optimizing the use of beds and hospitalization time is a constant concern, requiring the best possible management.

Considering surgical treatment, the American Academy of Orthopaedic Surgeons (AAOS)¹³ published a series of recommendations for the treatment of DFF in 2010. These recommend flexible intramedullary nailing as a therapeutic option for children aged five to 11 based on level III evidence and grade C recommendation. The use of rigid trochanteric intramedullary nailing, plate and screws, and flexible intramedullary nailing is recommended for individuals older than 11 based on level IV evidence and grade C recommendation. There is no evidence to recommend for or against the following situations: removal of surgical implants from asymptomatic patients after operative treatment of DFF (level IV evidence and inconclusive recommendation grade); outpatient physical therapy to improve function (level V evidence and inconclusive recommendation grade); and use of blocked plates versus unblocked plates for fracture fixation (level IV evidence and inconclusive recommendation grade).

 Considering these assumptions, we agree with the recommendations proposed by AAOS. ^15^ Our study showed that the mean ages for choosing blocked intramedullary nails were higher compared to the other groups in our casuistry (N = 16). The mean ages for using plates and screws were 12.17 years, 11.75 for using flexible nails, and 6.85 for external fixation. However, we did not adopt the conservative therapeutic approach for children older than five when possible. 

 We highlight the work by Luo Y et al., who advocate for the choice of flexible nails over plate and screw fixation in low-energy comminuted fractures, reporting advantages such as shorter surgical time, intraoperative bleeding, and hospitalization costs. ^5^ Of the nine cases observed in children aged ≤ 5 years, eight were treated with linear external fixation, and one with a flexible intramedullary nail. Our study demonstrated statistical significance in comparisons between fixation methods when considering the type of injury (Gustillo 3A open vs. closed) and fracture pattern (simple vs. complex), indicating that these variables are also invaluable factors in choosing the fixation method. 

 When judging the choice between external fixation and intramedullary nailing, the literature has not established a consensus. Our results, in pairwise evaluations, showed that the comparison between groups A and C did not present statistical significance (P < 0.001) when considering the age of the participants. Although our study did not demonstrate a positive association in the comparison between these groups and age, we believe that there is a tendency to assert that the use of external fixation may have superiority in patients with more distal or proximal fractures, older age, and body weight above 45 kg, compared to the use of flexible nails. ^18^

 In this study, there were six conversions in which external fixation was replaced by intramedullary nailing, four of which showed significant associated injuries (head, chest, cervical, or abdominal trauma). This is a common approach, as the implementation of external fixation is fast and effective, allowing for quick stabilization of the bone injury and enabling care for other systems and organs. ^19^ However, we emphasize that there are proponents of using external fixation definitively, especially in the pediatric population. ^13^ ^,^ ^20^ The more severe profile of patients undergoing external fixator therapy ^19^ ^,^ ^20^ could explain the higher number of individuals with open and/or complex fractures in Group D in our study. 

 Regarding complications, Group D showed a higher number of cases, with 50% showing complications, including one patient who developed non-union. The work of Lascombes et al. reports minimal complications, with one case of osteomyelitis, two refractures, five length discrepancies > 2 cm, and 12% skin irritation in over 350 treated fractures. They argue that the use of nails is superior to plates and external fixators. ^21^ Our study is in line with these findings, but our sample is limited, which may bias our results. 

 The length of hospitalization for our patients corresponded to the standard of care for polytraumatized individuals. A considerable rate of complications and adverse events, including mortality, is inherent in polytrauma cases among adolescents and pediatric patients. The severity of trauma is closely related to lower body weight and bone structure. In older children, thoracic and cranial trauma may be important predictive factors for outcomes in these patients, although extremities, the chest, and the abdomen are more frequently affected in this population. Besides the complications listed, we did not observe any fatal cases. ^22^

 We suggest that higher treatment costs are related to the severity of injuries. When comparing the financial demand, operative methods appear to be more advantageous when compared to conservative treatment, which could entail avoiding the risks of surgery. ^16^

The main limitation of our study is the small sample size, despite data collection spanning over 10 years (from January 2012 to December 2021). We consider the strong points to be the collection of data in a referral hospital for pediatric musculoskeletal injuries in the region. We believe that additional or multicenter studies could provide a larger sample size and offer more evidence on the subject.

## CONCLUSIONS

We conclude that the correct therapeutic choice in the management of diaphyseal femur fractures (DFF) in children and adolescents remains inconclusive in the literature, and it continues to be a topic of great relevance to the academic and scientific community. Our study confirms the association between age and fixation methods. Flexible intramedullary nails (INs) and external fixation were found to be the preferred options for patients of intermediate age, whereas locked INs, flexible INs, plates, and/or external fixation are prioritized for patients over 11 years old. The type and pattern of fractures are considered important factors in the choice of treatment. Despite complications being observed, the use of INs should not be discouraged. Finally, individual factors should be considered when choosing the best therapeutic approach. The functional outcome was satisfactory in 40 cases (97.6%) and unsatisfactory in one case (2.4%). Regarding fracture consolidation, we found one case (2.4%) of non-union and one case (2.4%) of malunion. In total, 39 cases (95.2%) achieved full and satisfactory healing of their injuries. Regarding complications, we noted that 50% of cases (N=3) occurred in Group D, with two major complications (joint motion limitation and pyoarthritis) and one categorized as minor (tendonitis). Moreover, in Group A, we found one minor complication (pin tract infection) and one major complication (malunion), whereas in Group C1, we found one minor complication (screw breakage in the nail).
